# Hepatitis B and C among women with illegal social behavior in Isfahan, Iran: Seroprevalence and associated factors

**Published:** 2011-05-01

**Authors:** Nazila Kassaian, Behrooz Ataei, Majid Yaran, Anahita Babak, Parisa Shoaei

**Affiliations:** 1 Infectious Diseases and Tropical Medicine Research Center, Isfahan University of Medical Sciences, Isfahan, IR Iran

**Keywords:** Women, Social behavior, Hepatitis B, Hepatitis C, Iran

## Abstract

**Background:**

In Iran, there is limited evidence on the prevalence of hepatitis B and C viruses (HBV and HCV) among females who engage in illegal sexual behavior.

**Objectives:**

To determine the prevalence of HBV and HCV infections and their associated factors in this population in Isfahan-Iran.

**Patients and Methods:**

In this cross-sectional study, 100 females who engaged in illegal sexual behavior during 2009-2010 in Isfahan were recruited from welfare to the DIC for women, and referrals were made among those who knew others who engaged in prostitution. Markers for HBV and HCV-Ab were measured by ELISA, and recombinant immunoblot assay was used for confirmation of HCV infection. Also, a questionnaire on demographics and prostitution-associated risk data in a face-to-face interview was completed for each participant. Chi-square and multivariate logistic regression models were used for data analysis.

**Results:**

Of the 100 samples collected, 91 were sufficient for testing. The mean age and time spent in sex work were 30.84 ± 9.34 years and 36 ± 28.5 months, respectively. HBsAg was detected in 1 (1.1%), anti-HBc in 4 (4.4%), anti-HBs in 60 (65.9%), and HCV Ab in 9 (9.9%) subjects. The evidence of vaccination was seen in 54 subjects (59.3%). There were no significant differences in the prevalence of HBV or HCV infection by estimated risk factors, and there was no independent risk factor for these infections.

**Conclusions:**

The high prevalence of HCV infection in this study indicates the need to implement preventive interventions for female sex workers and, perhaps more importantly, to involve their male clients.

## Background

Sexually transmitted infections (STIs) are a major public health problem among women, especially in developing countries [1]. STIs are usually concentrated in core groups, such as female sex workers (FSWs), who have a high number of partners and receive poor health care [2]. The evidence shows that females who engage in illegal sex activities experience high morbidity, which is related to their lifestyle [3][4]. Despite the recognition of prostitution as a public health issue, there is limited research on the prevalence of STIs and the marginalization that these groups always suffer, causing them to receive little attention [5]. Hepatitis B and C viruses (HBV and HCV), which have a mean prevalence of 3% globally, are major public health problems and can be transmitted between sexual partners [6]. There is some evidence that hepatitis B can be transmitted sexually, but such transmission of hepatitis C has remained controversial [7]. In Iran, the data on the extent to which these viruses spread among women who engage in illegal sexual activity are limited.

## Objectives

This study described the prevalence of HBV and HCV infection in a population of female sex workers in Isfahan, Iran, who, due to their profession, are at increased risk of acquiring and transmitting the disease. We also investigated the role of some risk factors for HBV and HCV infections in this population.

## Materials and Methods

### Setting and population

This study was conducted among females who engage in illegal sexual behavior who attended a health and social care drop-in center (DIC) for females and their friends who also engage in illegal sex activity during 2009-2010 in Isfahan, Iran. The DIC for women is a nongovernmental organization that is supervised by Isfahan University of Medical Sciences. This center provides care for women with high-risk behaviors, including IVDU and illegal sex activities. The eligibility criterion was a self-report of having sold sex for money within the previous 3 months.

### Design

With a 95% confidence interval, a sample size of 100 females with illegal sexual behavior was required to estimate a prevalence of HCV of approximately 7% [7][14][15][16]) with a precision of 5%. The participants were recruited through welfare referral to the DIC and by snowball sampling method, through referrals among people who knew others who sold sex for money. The study collected blood samples for HBV and HCV testing and demographic and prostitution-associated risk data in a face-to-face interview. All interviews were conducted by a social worker who had been working with this population. The questionnaire inquired about age, ethnicity, time in sex work, number of clients per week, condom use, drug use and the type, risk factors for HBV or HCV, frequency of marriage, having a temporary or permanent marriage, sex-related risk behavior, and history of incarceration.

### Ethical aspects

All eligible females with illegal sexual activities were requested to participate in the study after being given a brief description of the purpose and procedure of the study, after which they signed a written informed consent form. The participants enrolled in the study voluntarily and they did not receive any incentive for participation. The subjects' information was gathered anonymously, and participants could skip any question. This project was approved by the ethical committee of Isfahan University of Medical Sciences.

### Blood sampling and laboratory analysis

A 10-ml blood sample was obtained by venous puncture, and a serum sample was sent to the Isfahan Infectious Diseases Research Center Laboratory, where they were subjected to immunoenzymatic tests to detect HBV and HCV markers. Testing for antibody to HCV (HCV-Ab) was performed by ELISA (Diapro-Italy 3(rd) generation) and confirmed by recombinant immunoblot assay (Inno lopa-GMBH, Germany). HBV antibodies were assayed by ELISA (Diapro-Italy) for total hepatitis B core antibody (anti-HBc), hepatitis B surface antigen (HBs Ag), and hepatitis B surface antibody (anti-HBs). Tests were performed, and their cutoff points were defined per the manufacturer's instructions. HBV serological test results were classified as follows: Past immunity through infection (HBs Ag-negative, anti-HBc-positive, and anti-HBs-positive), Acute hepatitis B (HBs Ag-positive, anti-HBc-positive, and anti-HBs-negative), and serological evidence of vaccination (anti-HBs-positive, HBs Ag-negative, and anti-HBc-negative).

### Statistical Analysis

Statistical analysis was performed with SPSS-15 for Windows (InC, Chicago, IL). Prevalence of viral hepatitis (HBV, HCV) was estimated using descriptive statistics. Univariate associations between risk factors and HCV and HBV seropositivity were evaluated by chi-square test. Adjusted odds ratios (ORs) were calculated by including factors in the multivariate model (logistic regression), and P ≤ 0.05 was considered statistically significant in the overall analysis.

## Results

Of 100 samples that were collected, 91 were sufficient for testing. Their main characteristics are shown in Table 1. The mean age and time in sex work were 30.84 ± 9.34 years and 36 ± 28.5 months, respectively. Among participants who answered the question on sexual behavior (No. = 81), 21 (27.6%), 73 (96.1%), and 26 (34.2%) practiced oral, vaginal, and anal sex with their clients, respectively. Continuous and intermittent condom use by sexual practice was reported by 44 (48.35%) and 15 subjects (16.48%), respectively. HBs Ag was detected in 1 subject (1.1%), anti-HBc in 4 (4.4%), anti-HBs in 60 (65.9%), and HCV-Ab in 9 (9.9%). Evidence of vaccination was seen in 54 subjects (59.3%). There were no significant differences in the prevalence of HBV or HCV infection by estimated risk factors using the univariate model (Table 2). In the multivariate model, there was no independent risk factor for HCV infection (Table 3). Due to the low prevalence of HBV infection in our subjects, multivariate logistic regression could not be used to adjust HBV risk factors.

**Table 1:** Main characteristics of study participants (No. = 91)

**Variable**	**No.**	**%**
**Education**		
**None or primary studies **	23	25.3
**Secondary**	24	26.4
**Diploma**	28	30.7
**University**	16	17.6
**Ethnicity **		
**Iranian **	89	97.8
**Afghani**	2	2.2
**Number of Clients per week **		
**1-2**	23	25.3
**3-4**	42	46.1
**5 ≤**	26	28.6
**Frequency of permanent marriage **		
**0**	16	17.5
**1**	57	63
**2**	16	17.5
**3**	2	2
**Frequency of temporary marriage**		
**0**	57	63
**1**	14	15.4
**2**	14	15.4
**3**	2	2.2
**4-6**	4	4
**Drug Addiction **		
**No**	31	39
**IVDU**	24	19
**Other types**	63	57

**Table 2:** Risk Estimating for HBV and HCV infections (univariate analysis)

**Variables a**	**HBV Infection OR b**(95% CI)	**HCV Infection OR** (95% CI)
**Tattooing **	0.5 (0.051-6.7)	0.3 (0.06-1.5)
**Transfusion**	1.25 (0.1-14.5)	0.7 (0.1-3.5)
**Surgery**	1.8 (0.15-20.7)	1.1 (0.3-4.5)
**Cupping **	4 (0.35-53.8)	2.6 (0.46-15.3)
**Prison history **	0.87 (0.76-10.09)	2.4 (0.6-9.96)
**Drug addiction **	1.27 (0.1-14.7)	0.56 (0.046-1.7)
**Intravenous Drug Abuse **	1.6 (0.1-18.6)	2.9 (0.7-12.3)
**Sexual behavior**		
**Oral sex**	1.3 (0.1-15.43)	0.3 (0.03-2.5)
**Vaginal Sex**	0.96 (0.9-1)	0.95 (0.9-1)
**Anal sex**	0.96 (0.08-11.1)	0.6 (0.05-1.7)
**Using Condom **	0.62 (0.05-7.4)	0.25 (0.05-1.2)

^a^ 0 = No, 1 = Yes;Reference, category = No

^b^ OR = Odds Ratio

**Table 3:** Multivariate Logistic Regression of possible risk factors for HCV infection

**Variable a**	**Adjusted ORb**(95% CI)	**p-value**
**Age** (years)	0.96 (0.87-1.06)	0.5
**Temporary marriage**	0.47 (0.07-3.1)	0.4
**Transfusion a**	2.79 (0.38-20.59)	0.3
**Surgery a**	0.35 (0.05-2.41)	0.3
**Cupping a**	0.33 (0.03-2.78)	0.3
**Prison history a**	0.5 (0.09-2.65)	0.4
**IVDU a**	0.17 (0.02-1.44)	0.1
**Time in sex work**	0.97 (0.98-1.01)	0.16

^a^ 0 = No, 1 = Yes;Reference, category = No

^b^ OR = Odd Ratio

## Discussion

To our knowledge, this is the first survey that has provided local, representative estimates of the seroprevalence of viral hepatitis B and C in females who engage in illegal sex behavior in Isfahan-vital information that provides insight into disease burden and opportunities for prevention. The prevalence of past infection and acute hepatitis B in our sample (4.4% and 1.1%, respectively) did not different significantly from the prevalence of the infection in the general population of Isfahan (4.2% and 1.3%, respectively) [8]. In a survey in Tehran-Iran, in a female population who engaged in illegal social behavior, 196 females who were arrested by police, 79% of whom had a history of prostitution, were positive for HBs Ag and HCV Ab, and 1.5% was HBs Ag-positive [7], which is similar to our results. In other parts of the world, the prevalence of the infection differs in prostitutes, which reflects the health and social status of those regions. In Nigeria, the overall HBV prevalence among FSWs is 17.1% [9]. In a city (Surat city) in India, HBV (HBs Ag) was detected in 3.33% of FSWs [1]. In preto-Sao Paulo, Brazil, HBs Ag and anti-HBc were detected in 0.7% and 22.3% of female prostitutes, respectively [10].

In a recent study, serological evidence of vaccination was seen in 59.3%, which is much higher than in the general population of Isfahan. In Nokhodian et al. [8], 12.1% of the total population of Isfahan was positive for anti-HBs and negative for HBs Ag and anti-HBc-evidence of vaccination. This finding shows that our subjects follow the WHO recommendations for universal vaccination against HBV as the best strategy for reducing the risk of HBV infection, and it may be the cause of the low prevalence of HBV infection in them. Regarding the prevalence of hepatitis C, a systematic review from 2009 calculated that the HCV infection prevalence rate in the general population in Iran was 0.16% (95% CI: 0%-0.59%) [11]. Thus, the high prevalence of HCV infection (9.9%) among our subjects is the other principal finding of this study.

Although we were unable to ascertain the source of HCV in these women (the association between positivity and the use of injectable drugs and other risk factors was not determine by univariate and multivariate analysis), our study suggests that sexual transmission may be important in the spread of HCV, whereas many studies have failed to carefully exclude the acquisition of HCV from nonsexual sources [11][12]. The high rate of HCV in the prostitutes in our study contrasts that of blood donors in Iran (0.12%) [13].

In Jahani et al. [7], HCV seropositivity was seen in 3.1% of women who were arrested by police in Tehran, Iran. In a study on prostitutes from Spain who denied intravenous drug abuse, 5.78% was seropositive for HCV [14]. Among FSWs in the city of Santos, Saopaulo state, Brazil, the prevalence of HCV antibodies was 10.9% [15]. In a study in the Congo, the overall prevalence of anti-HCV was 6.6% among female commercial sex workers [16]. The high prevalence of HCV infection in this study indicates the need to implement preventive interventions for FSWs and, perhaps more importantly, to involve their male clients.

### Limitations

We found no significant association between any risk factor and HBV or HCV infection, which could be due to random error due to the small sample size. Also, the sample of this study was not necessarily representative of women who engage in illegal social behavior in Isfahan, Iran, because selection bias due to the approach of convenience sampling in a health and social care setting (DICs) might have occurred. In Iran, the presence of prostitution is systematically denied, and therefore, the investigation of sex workers is very difficult, causing the limited sampling and sample size in this study. The data on hepatitis infections and related complications among FSWs in Iran are limited, which means that the burden of these diseases is substantially underestimated. Therefore, further investigation on viral hepatitis infections and sexual practice and adopting protective measures against such infections are paramount in developing intervention programs.
